# Ophthalmic complications including retinal detachment in hyperimmunoglobulinemia E (Job's) syndrome: Case report and review of literature

**DOI:** 10.4103/0301-4738.55076

**Published:** 2009

**Authors:** Vipul Arora, Usha R Kim, Hadi M Khazei, Shivayogi Kusagur

**Affiliations:** Orbit, Oculoplasty and Oncology Clinic, Aravind Eye Hospital and Postgraduate Institute of Ophthalmology, Madurai, Tamil Nadu, India

**Keywords:** Blepharitis, Job's syndrome, retinal detachment

## Abstract

Hyperimmunoglobulinemia E (Job's) syndrome is characterized by markedly increased levels of immunoglobulin E, recurrent cutaneous and systemic pyogenic infections, atopic dermatitis, and peripheral eosinophilia. Although ocular involvement in Job's syndrome is rare, there are reports of keratoconus, staphylococcal chalazia with blepharitis, and *Candida* endophthalmitis by various authors. We present the first case report of retinal detachment with complicated cataract in Job's syndrome.

Hyperimmunoglobulinemia E (HIE) or Job's syndrome is an immunodeficiency that was first described by Davis *et al*.[1] The name Job was chosen with reference to the Book of Job 2:7: “So went Satan forth from the presence of the Lord, and smote Job with some boils from the sole of his feet unto his crown.” Job's syndrome and Buvkley's syndrome are subsets of the HIE syndrome. Elevated levels of serum immunoglobulin E (IgE) with values reaching >2000 IU (normal<200 IU) is the characteristic manifestation, which is due to defective T suppressor cell function. Herein we report a case with characteristic manifestation but with an additional, not yet reported finding of retinal detachment in Job's syndrome.

## Case Report

**Figure 1:**
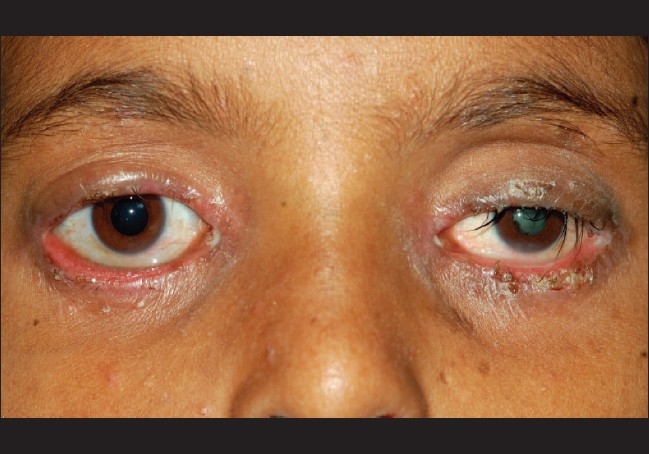
Presentation of patient with bilateral cicatrical ectropion with ulcerative blepharitis and complicated cataract in the left eye

**Figure 2:**
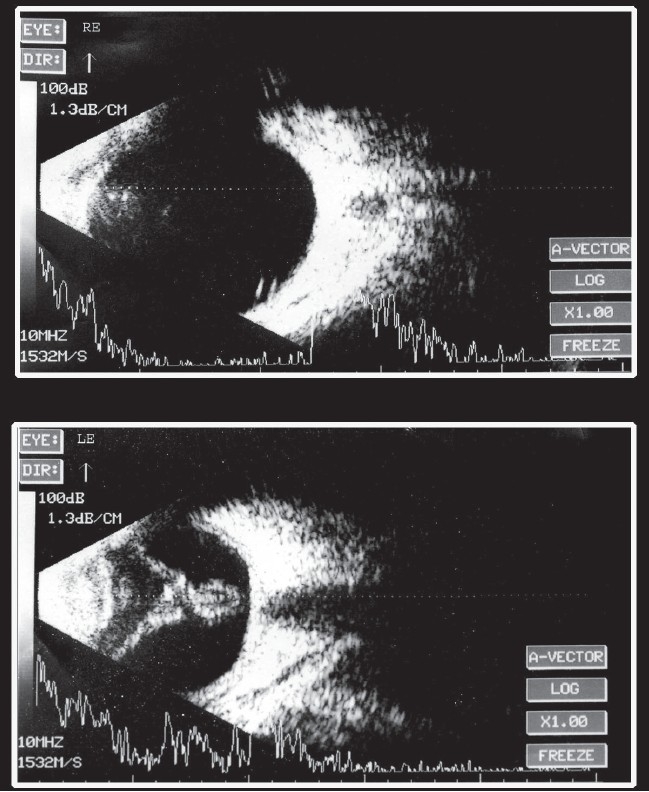
Ultrasonogaphy B-scan of both eyes showing no abnormality in the right eye whereas the left eye shows presence of longstanding closed funnel retinal detachment

A 15-year-old male child had watering, irritation, and itching in both eyes of two years duration. He noticed dimness of vision in his left eye one year ago. Patient denied any history of trauma in the left eye. On presentation patient had visual acuity of 20/20 in the right eye and perception of light with inaccurate projection in the left eye. External ocular examination revealed bilateral cicatrical ectropion with infective ulcerative blepharitis and lagophthalmos [Fig. 1]. In the left eye, the patient had an afferent pupillary defect with complicated cataract and no signs of uveitis. Intraocular pressure was 15 mm Hg in the right eye and 4 mm Hg in the left eye. B-scan ultrasonography in the left eye showed an hyperechoic membrane attached to the optic disc which could be appreciated on reducing the gain. This was suggestive of a longstanding closed funnel retinal detachment [Fig. 2]. Systemic examination revealed nail dystrophy, hyperextensibility of joints and hyperpigmented papulo-vesicular skin lesions suggestive of scabies over the groin. Blood investigation showed raised eosinophil levels. Serum IgA was within normal range, 3.53/IU/ml (normal 0.90-4.50g/l), but Serum IgE was 7375.2IU/ml (normal 1.5-378.0 IU/ml), which exceeded the normal value by 20 times. Human immunodeficiency virus (HIV) screening was negative. On the basis of ocular findings, systemic manifestations, and raised serum IgE levels, the patient was diagnosed as a case of left eye rhegmatogenous retinal detachment and complicated cataract associated with features of HIE syndrome. Patient was treated with azithromycin (250 mg) once a day for one week to prevent staphylococcal infection secondary to scabies, permethrin solution 5% for the groin lesions along with antihistamine tablets and topical antibiotic ointment in both eyes.

## Discussion

The HIE syndrome is an autosomal dominant disease with variable expressivity; many patients show a partial phenotype. The responsible gene has been mapped to Chromosome 4q, although it has not yet been identified.[2] Cell-mediated immunity is often abnormal in patients with HIE; the most common functional defects in host resistance are defective suppressor T cell function and defective neutrophilic chemotaxis.

Patients with HIE syndrome generally present with chronic and intense pruritis associated with widespread eczematous dermatitis which occasionally involve the eyelids. This pruritis is attributed to intradermal mast-cell histamine release triggered by the elevation of IgE.[3] Staphylococcal skin infection is common and manifests in the form of impetigo, furunculosis, paronychia or cellulitis. The characteristic feature of staphylococcal infection in Job's syndrome is abscess formation without the anticipated degree of erythema and warmth. Pulmonary bacterial pneumonia and emphysema are the most frequent systemic infections and may result in pneumatoceles that become the nidus for further bacterial and fungal infection. The most common infecting organisms are *Staphylococcus aureus* and *Hemophilus influenzae*. Job's syndrome has also been associated with scoliosis in 76% of adult patients and hyperextensibility of joints in 68% of patients.[2] Peripheral blood eosinophilia may be marked, reaching levels of 50–60%. Serum IgE levels are consistently high (more than 10 times the upper limit of normal), even in infancy.

Kim *et al*.[4] reported a case of bilateral keratoconous in a 28-year-old male suffering from Job's syndrome. Although this association was of unknown etiology, it is important to consider this in HIE patients. In our patient, corneal topography of both eyes failed to demonstrate any ectasia. Destafeno *et al*.[5] reported a case of a 16-year-old female with recurrent chalazia associated with *Staphylococcus aureus* along with other manifestations of this syndrome. Frohn *et al*.[6] and Orhan *et al*.[7] have reported cases of corneal ulceration and perforation in patients with Job's syndrome presumably caused by *Staphylococcus aureus* despite aggressive systemic and topical antibiotic therapy. Haslett *et al*.[8] have reported a case of endogenous *Candida albicans* endophthalmitis in a 24-year-old female.

This is the first case report of retinal detachment with complicated cataract in HIE or Job's syndrome in a young male. Our patient had no history of trauma or signs of uveitis in the involved eye. We speculate that the etiology and pathogenesis of retinal detachment in case of Job's syndrome closely resembles with that of atopic dermatitis, which is an IgE-mediated hypersensitivity reaction. Atopic dermatitis patients usually have multiple breaks that occur in the peripheral retina and vitreous base which may lead to rhegmatogenous retinal detachment and giant retinal breaks.[9] The etiology for these breaks is attributed to vigorous rubbing of eyes due to intense itching, which could also be a probable cause of retinal detachment in our case. This detachment is non-inflammatory in nature as photoreceptors' outer segments have been detected in aqueous of eyes of patients with atopic dermatitis with retinal detachment.[9]

Management in our case was only observation as patient had longstanding closed funnel retinal detachment with hypotony of the globe. Patients with Job's syndrome should be carefully evaluated for detailed posterior segment examination and in cases where media opacity is present, B-mode ultrasonography should be done. This aids in early detection and treatment of retinal detachment, thereby reducing ocular morbidity and preventing visual loss.
